# Differences in clinical valve size selection and valve size selection for patient-specific computer simulation in transcatheter aortic valve replacement (TAVR): a retrospective multicenter analysis

**DOI:** 10.1007/s10554-019-01688-5

**Published:** 2019-09-12

**Authors:** Nahid El Faquir, Giorgia Rocatello, Zouhair Rahhab, Johan Bosmans, Ole De Backer, Nicolas M. Van Mieghem, Peter Mortier, Peter P. T. de Jaegere

**Affiliations:** 1 Department of Cardiology, Thoraxcenter, Erasmus Medical Center, ‘s-Gravendijkwal 230, 3015 CE Rotterdam, The Netherlands; 2 IBiTech-bioMMeda, Ghent University, Ghent, Belgium; 3 Department of Cardiology, University Hospital Antwerp, Antwerp, Belgium; 4 Department of Cardiology, Rigshospitalet University Hospital, Copenhagen, Denmark; 5 FEops NV, Ghent, Belgium

**Keywords:** Aortic stenosis, TAVR, Computer simulation

## Abstract

Valve size selection for transcatheter aortic valve replacement (TAVR) is currently based on cardiac CT-scan. At variance with patient-specific computer simulation, this does not allow the assessment of the valve-host interaction. We aimed to compare clinical valve size selection and valve size selection by an independent expert for computer simulation. A multicenter retrospective analysis of valve size selection by the physician and the independent expert in 141 patients who underwent TAVR with the self-expanding CoreValve or Evolut R. Baseline CT-scan was used for clinical valve size selection and for patient-specific computer simulation. Simulation results were not available for clinical use. Overall true concordance between clinical and simulated valve size selection was observed in 47 patients (33%), true discordance in 15 (11%) and ambiguity in 79 (56%). In 62 (44%, cohort A) one valve size was simulated whereas two valve sizes were simulated in 79 (56%, cohort B). In cohort A, concordance was 76% and discordance was 24%; a smaller valve size was selected for simulation in 10 patients and a larger in 5. In cohort B, a different valve size was selected for simulation in all patients in addition to the valve size that was used for TAVR. The different valve size concerned a smaller valve in 45 patients (57%) and a larger in 34 (43%). Selection of the valve size differs between the physician and the independent computer simulation expert who used the same source of information. These findings indicate that valve sizing in TAVR is still more intricate than generally assumed.

## Introduction

Transcatheter aortic valve replacement (TAVR) is an accepted treatment in patients with severe aortic stenosis at a high or intermediate operative risk [1–5]. At present, different valve types and sizes are available allowing optimal transcatheter valve performance and clinical outcome in a wide range of patients [5–8]. Availability of different valves and sizes implies that choosing the valve that best fits the individual patient has become more challenging in particular as outcome of TAVR depends among others on device-host interaction. Currently, computed tomography (CT) scan of the heart is the standard method and recommended for selection of the valve size [9]. Yet, this does not allow the prediction of the mechanical interaction and precise outcome between the device and host. For that purpose, a dedicated computer simulation model has been developed and validated to predict case-by-case calcium displacement, presence and severity of aortic regurgitation (AR) and conduction disturbances post TAVR [10–13]. The purpose of this study was to compare transcatheter valve size selection between the physician for TAVR and the independent expert for the purpose of patient-specific computer simulation.

## Patients and methods

### Study population

The study population consisted of 141 patients who had undergone TAVR with the self-expanding Medtronic valve (CoreValve [MCS] or Evolut R) because of native tricuspid aortic stenosis and in whom patient-specific computer simulation was performed for the assessment and prediction of valve performance. The information of the computer simulation was not available for clinical application. The valve size selected for the clinical implantation (TAVR) was decided by the physician based upon pre-procedural multi-slice computed tomography (MSCT) as previously described [14]. In all patients and participating centers dedicated software (i.e., 3 Mensio, Pie Medical Imaging BV, Maastricht, the Netherlands) was used for quantitative analysis of the aortic root [15].

All patients provided written informed consent for TAVR and data collection. Clinical data was extracted from local databases. The study was conducted in accordance with the principles of the Declaration of Helsinki and did not fall under the scope of the Medical Research Involving Human Subjects Act per EMC Institutional Review Board (MEC nr. 2019-0442).

*Patient-specific computer simulation* was performed by an independent institution (FEops, Ghent, Belgium) by first creating a virtual model of each patient’s anatomy by segmentation and 3D reconstruction of the aortic root *(including the calcified native leaflets and outflow tract [LVOT])* based on pre-procedural anonymized MSCT *(Mimics Software, Materialise, Leuven, Belgium)* [10–13]. All steps performed during TAVR were respected during simulation such as eventual pre- or postdilatation and valve type (i.e., *MCS, Evolut R*) but not valve size (*Abaqus/Explicit finite element solver, Dassault Systèmes, Paris, France*). The valve size used during simulation was decided by the independent simulation expert using the aortic annulus dimensions and the manufacturer’s matrix of sizing. As the latter contains precise cut-off values for each MCS and Evolut R valve size, a margin of ± 2% was used for each cut off value (i.e., grey zone of valve size selection). In case a patient had an aortic annulus dimension that falls within the grey zone, 2 sizes were simulated. During simulation, 3 levels of depth of implantation were executed; 2 mm below the annulus (high implant), 5 mm below the annulus (medium depth of implantation) and 8 mm below the annulus (low implant).

Outcome measures of the simulation were: (1) coronary obstruction, (2) aortic regurgitation (AR) and (3) contact pressure of the frame within a predefined area below the annulus [10–13]. Coronary obstruction was defined by measuring the distance between the closest native aortic leaflet calcium nodule and the center of the left and right coronary ostium post TAVR (i.e., coronary obstruction present if distance: 0 mm) [10]. Severity of AR post TAVR was measured using computational fluid dynamics and was expressed in ml/s. A cut-off value of 16.0 ml/s has been reported to correspond to ≥ moderate AR [11]. Contact pressure exerted by the frame in the region of the LVOT hosting the conduction tissue was expressed as maximum contact pressure (MPa) and area (i.e., contact pressure index, %). Cut-off values of respectively 0.39 MPa and 14% were earlier found to correlate with the occurrence of new left bundle branch block (LBBB) or high degree atrio-ventricular block (high degree AVB) [13].

Clinical outcome measures (after TAVR) were: (1) coronary obstruction defined by TIMI 0 flow during contrast angiography immediately post TAVR, (2) AR assessed by transthoracic echocardiography before discharge [VARC-2 criteria, 16] and (3) LBBB or high degree AVB (i.e., Mobitz II or 3rd degree AV block) defined by 12-lead ECG before discharge [17].

### Statistical analysis

The main analysis consisted of the assessment of the agreement between the valve size selected for simulation and TAVR and, the comparison between the predicted (simulation) and clinical (TAVR) outcomes, namely (1) coronary obstruction, (2) AR and (3) new LBBB or high degree AVB in relation to maximum contact pressure and area after TAVR. For the purpose of this study, the simulation-derived outcome measures at a medium depth of implantation were used.

Normality of distributions was tested by the Kolmogorov–Smirnov test. Continuous variables are shown as mean ± standard deviation (SD) or medians [interquartile ranges (IQR) 25–75%] as appropriate. Categorical variables are expressed as frequencies and percentages. Comparison between predicted and observed coronary obstruction and AR post TAVI (≥ grade 2) was made by means of the McNemar’s test.

## Results

### Study population

The baseline demographic and procedural characteristics of the 141 patients are summarized in Table 1. The median age was 82 (78–85) years and 50% were male. In 115 patients (82%) a MCS valve was implanted while 26 (18%) received an Evolut R valve. With respect to valve size, a 29 mm valve was implanted in 92 patients (62%), a 26 mm in 40 (28%) and a 31 mm in 9 (6%). In all patients some degree of oversizing was observed, 18 ± 7% when using the perimeter and 20 ± 7% when using the mean diameter.

**Table 1:** Baseline, MSCT and procedural characteristics

	Entire cohort
	n = 141
Baseline	
Age (years)	82 (78–85)
Gender (male)	70 (50)
Left bundle branch block	15 (11)
Right bundle branch block	6 (4)
Pacemaker	15 (11)
Multi-slice computed tomography	
Annulus	
Minimum diameter (mm)	20.5 ± 2.1
Maximum diameter (mm)	26.8 ± 2.3
Mean diameter (mm)	23.6 ± 1.9
Perimeter (mm)	75.5 ± 5.9
Perimeter derived diameter (mm)	24.0 ± 1.9
Area (mm^2^)	428.9 ± 66.2
Area derived diameter (mm)	23.3 ± 1.8
Procedural and sizing	
Predilatation	122 (87)
Predilatation balloon nominal/mean annulus diameter × 100 (%)	90 ± 9
Valve type	
Medtronic CoreValve	115 (82)
Medtronic Evolut R	26 (18)
Valve size	
26	40 (28)
29	92 (62)
31	9 (6)
Valve size/mean annulus diameter × 100 (%)	120 ± 7
Valve perimeter/perimeter of the annulus × 100 (%)	118 ± 7
Depth of implantation non coronary cusp	7.2 ± 3.4
Depth of implantation left coronary cusp	8.3 ± 4.1
Mean depth of implantation	7.7 ± 3.6
Postdilatation	13 (9)
Postdilatation balloon nominal/mean annulus diameter × 100 (%)	105 ± 5

Values are expressed in median (interquartile range), n (%) or mean ± SD

### Findings

In 62 out of the 141 patients (44%) one valve size was simulated (cohort A), 2 valve sizes were simulated in 79 patients (56%, cohort B). In cohort A (n = 62), concordance (i.e., *same valve size selected for simulation and TAVR*) was 76% (47 patients). Discordance was observed in 15 patients (24%, Table 2); a smaller valve was selected for simulation in 10 patients and a larger in 5. In cohort B (n = 79, Table 3), a different valve size was selected for simulation in all patients in addition to the valve size that was used for TAVR; a smaller valve was selected for simulation in 45 patients (57%) and a larger in 34 (43%). This means that overall true concordance (*same valve size selection for simulation and TAVR by independent expert or physician*) was observed in 33% of patients, true discordance in 11% and ambiguity (i.e., *two valve sizes selected for simulation and thus* a priori *considered eligible for TAVR*) in 56% of patients. In case of discordance and ambiguity, a smaller valve was used for simulation in 39% of patients and a larger in 28%. In case of discordant valve size selection in cohort A (n = 15), the degree of oversizing was 18 ± 7% when using the perimeter and 20 ± 7% when using the mean diameter. It was 17 ± 8 and 15 ± 7%, respectively in cohort B (different valve size used for simulation).

**Table 2:** Outcome in cohort A (simulation of one valve size)

	Concordant clinical valve size and simulated valve size n = 47			Discordant clinical valve size and simulated valve size n = 15		
	Predicted	Observed	p-value	Predicted	Observed	p-value
Coronary obstruction	0	0	–	0	0	–
Aortic regurgitation (ml/s)	14.0 (6.6–24.4)	–		16.6 (11.3–23.7)	–	
Aortic regurgitation ≥ grade 2	17 (36)	4 (9)	0.002	9 (60)	3 (20)	0.07
Maximum contact pressure	0.44 ± 0.25	–		0.33 ± 0.24	–	
Maximum contact pressure ≥ 0.39 MPa	24 (51)	–		4 (27)	–	
Contact pressure index	0.21 ± 0.10	–		0.14 ± 0.10	–	
Contact pressure index ≥ 14%	35 (75)	–		5 (33)	–	
New LBBB or high degree AVB	–	25 (53)		–	7 (47)	

Values are expressed in n (%), median (interquartile range) or mean ± SD

*LBBB* left bundle branch block, *High degree AVB* high degree atrio-ventricular block

**Table 3:** Outcome in cohort B (simulation of two valve sizes)

	Concordant clinical valve size and simulated valve size n = 79			Comparison between clinical valve size and additional (discordant) simulated valve size n = 79		
	Predicted	Observed	p-value	Predicted	Observed	p-value
Coronary obstruction	0	0	–	0	0	–
Aortic regurgitation (ml/s)	12.2 (5.1–22.1)	–		15.4 (7.5–27.5)	–	
Aortic regurgitation ≥ grade 2	33 (42)	16 (20)	0.007	38 (48)	16 (20)	0.001
Maximum contact pressure	0.40 ± 0.24	–		0.41 (0.26–0.62)	–	
Maximum contact pressure ≥ 0.39 MPa	36 (46)	–		39 (49)	–	
Contact pressure index	0.18 ± 0.11	–		0.19 (0.12–0.27)	–	
Contact pressure index ≥ 14%	49 (62)	–		50 (63)	–	
New LBBB or high degree AVB	–	42 (53)		–	42 (53)	

Values are expressed in n (%), median (interquartile range) or mean ± SD

*LBBB* left bundle branch block, *High degree AVB* high degree atrio-ventricular block

#### Outcome cohort A

In correspondence with the clinical observation, simulation did not predict any case of coronary obstruction (Table 2). In case of discordant valve size selection, the simulation predicted a higher degree of AR post TAVR [16.6 (11.3–23.7) ml/s] and (*based upon the recently validated cutoff*-*value of AR grade *≥ *2*) a higher prevalence of AR ≥ grade 2 (60%) in comparison with concordant valve size selection [14.0 (6.6–24.4) ml/s and 36%), albeit that in both situations the predicted AR exceeded the observed AR. Predicted AR ≥ grade 2 was significantly higher compared to observed in case of concordant valve size selection (p = 0.002, Table 2). The predicted maximum contact pressure and contact pressure index were lower in case of discordant valve size selection (Table 2). The prevalence of clinical (i.e., observed) new LBBB and high degree AVB after TAVR were similar between con- and discordant valve size selection.

#### Outcome cohort B

Similar to cohort A, coronary obstruction was neither predicted nor observed (Table 3). In case of simulation of a valve size different from TAVR, the simulation predicted a higher degree of AR post TAVR [15.4 [7.5–27.5]) ml/s] and a higher prevalence of AR ≥ grade 2 (48%) in comparison with concordant valve size selection [12.2 (5.1–22.1) ml/s and 42%]. Predicted AR ≥ grade 2 was higher than observed in both concordant and discordant valve size selection (respectively p = 0.007 and p = 0.001). No difference in predicted maximum contact pressure and contact pressure index were seen when using the same or different valve size for simulation (Table 3).

## Discussion

When comparing valve size selection by the physician (*using MSCT and dedicated software for quantitative analysis of the aortic root*) with valve size selection for patient-specific computer simulation, we found true concordance in 33% of the patients, true discordance in 11% and ambiguity in 56%. Moreover, a smaller valve was selected for simulation in 39% of the patients and a larger in 28%. These findings indicate that valve size selection in TAVR still is more intricate than generally assumed even when using MSCT that is the standard imaging modality for TAVR planning including valve size selection [9].

These findings need to be interpreted against the following; firstly, the physician generally adheres to the cut-off values for sizing proposed by the manufacturer’s matrix of sizing albeit with some degree of oversizing while a margin of 2% at each cut-off value in two directions was used for the selection of valve size(s) for computer simulation. This margin of which its value (i.e., 2%) was arbitrarily chosen was introduced as the application of strict cut-off criteria in clinical practice is often not realistic or preferable and lacks a sound pathophysiologic basis. The use of such a margin or grey zone may explain the observation that a smaller valve size was used for simulation in 39% of the patients and a larger in 28% in the present analysis.

There are distinct differences between clinical valve size selection (*physician*) and valve size selection based upon computer simulation. In the former, the physician selects the valve size upon the quantitative assessment of the dimensions of the aortic root while in the latter–that starts with MSCT analysis–the effects of the device-host interaction (e.g*., coronary obstruction, AR post TAVR and conduction abnormalities*) are taken into account. The device-host interaction is based upon the integration of the dimensions of the device and host and, their biomechanical properties. Tissue biomechanical properties are derived from experimental data subsequently refined during so-called training of the computer model during validation studies [10–13]. Biomechanical properties of the frame are derived from standard in vitro testing of the stress–strain relationship [18]. Of note, the biomechanical properties of the nitinol frame of the herein used valves are such that its hysteresis loop has a plateau (i.e., *no change in radial force during a certain phase of frame compression or expansion*) with some degree of overlap between two adjacent valve sizes. This implies that–when a physician decides to oversize to ensure proper anchoring and to avoid PVL–such a strategy does not necessarily translates into a higher contact pressure exerted by the frame on the LVOT and consequently new conduction abnormalities. This is supported by the observation that the predicted maximum contact pressure and contact pressure index were similar when using the same or different valve size for simulation in cohort B. However, a higher predicted maximum contact pressure and contact pressure index is seen in concordant cohort A versus discordant cohort A which we believe is because majority (10/15) of simulations in discordant valve size selection was performed with a smaller valve compared to clinical practice. A smaller implanted valve is expected to result in less contact pressure. With regard to AR ≥ grade 2, a higher frequency was predicted than observed in cohort A (concordant) and cohort B (concordant and discordant) which may be the result of AR post TAVR quantification by means of echocardiography based on the VARC 2 method since it is known that echocardiography is inferior to magnetic resonance imaging (MRI) for the assessment of AR and underestimates the actual degree of AR [19].

The selection of the valve size (and type) that best fits the individual patient is mandatory as a patient-specific or -tailored approach ensures maximum safety and efficacy. This is in particular important with the increasing number of sizes and types of valves that are and will be available in clinical practice in combination with the fact that the aortic root differs from patient to patient. We acknowledge that in this analysis, we concentrated on one single valve type (i.e., the self-expanding MCS and Evolut R) and did not include the balloon-expandable and mechanically expanding valves. Yet, conceptually the role of patient-specific computer simulation in clinical practice is self-explanatory. This, however, needs to be proven by appropriately designed studies such as RCT (physician vs. simulation driven valve size and type selection) and implies that the software keeps track with the rapid development of novel valve technologies and sizes. Given the rapid development of valve technologies that effectively address the vexing issues of TAVR (AR in particular), patient-specific computer simulation may have in particular a clinical value in cases of uncertainty in valve size selection for instance due to the strict sizing criteria proposed by the manufacturer in combination with observer variability in MSCT analysis. It may especially have a clinical added value in the selection of the valve type that best fits the individual patient.

## Limitations

This study concerns an exploratory analysis of the comparison between valve size selection between the physician for TAVR and the independent expert for the purpose of patient-specific computer simulation. The study design consisted of a retrospective analysis which may introduce bias. Predicted outcomes represent the situation directly post TAVR and no conclusions on long term can be drawn based on this study. In addition to the limitations discussed above, the sample and the fact that only three centers participated need to be considered and affect generalizability.

## Conclusion

Selection of the size of the self-expanding valve by the physician for TAVR and the independent expert for patient-specific computer simulation differs substantially. Concordance was found in only 33% of the patients, true discordance in 11% and ambiguity in 56%. A smaller valve was selected for simulation in 39% of the patients and a larger in 28%. These findings indicate that valve sizing in TAVR is still more intricate than generally assumed.
